# Natural deep eutectic solvent-based microextraction for mercury speciation in water samples

**DOI:** 10.1007/s00216-023-04610-0

**Published:** 2023-03-06

**Authors:** Laura Ripoll, Javier Rayos, Miguel Ángel Aguirre, Lorena Vidal, Antonio Canals

**Affiliations:** Departamento de Química Analítica, Nutrición y Bromatología e Instituto Universitario de Materiales, Universidad de Alicante, P.O. Box 99, Alicante, 03080 Spain

**Keywords:** Natural deep eutectic solvent, Environmental samples, Green analytical chemistry, Microextraction, Mercury speciation

## Abstract

A new natural deep eutectic solvent (NADES)-based analytical method for mercury speciation in water samples is presented. A NADES (i.e., decanoic acid:DL-menthol in a molar ratio of 1:2) is used as an environmentally friendly extractant for separation and preconcentration using dispersive liquid–liquid microextraction before LC-UV–Vis. Under optimal extraction conditions (i.e., NADES volume, 50 µL; sample pH, 12; volume of the complexing agent, 100 µL; extraction time, 3 min; centrifugation speed, 3000 rpm; and centrifugation time, 3 min), the limit of detection values were 0.9 µg L^−1^ for the organomercurial species and 3 µg L^−1^ for Hg^2+^, which had a slightly higher value. The relative standard deviation (RSD, *n* = 6) has been evaluated at two concentration levels (25 and 50 µg L^−1^) obtaining values for all the mercury complexes within the range of 6–12% and 8–12%, respectively. The trueness of the methodology has been evaluated using five real water samples from four different sources (i.e., tap, river, lake, and wastewater). The recovery tests have been performed in triplicate obtaining relative recoveries between 75 and 118%, with RSD (*n* = 3) between 1 and 19%, for all the mercury complexes in surface water samples. However, wastewater sample showed a significant matrix effect (recoveries ranged between 45 and 110%), probably due to the high amount of organic matter. Finally, the greenness of the method has also been evaluated by the analytical greenness metric for sample preparation (i.e., AGREEprep).

**Graphical Abstract:**

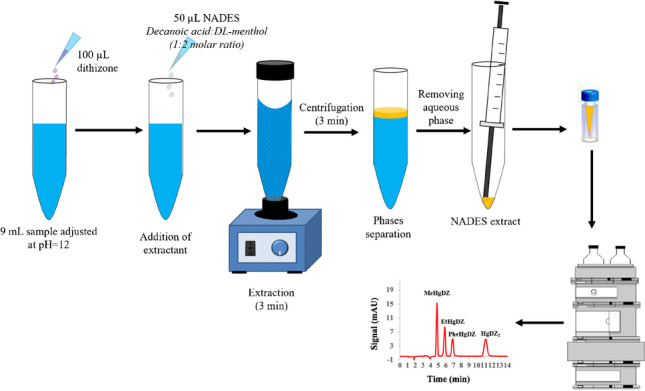

**Supplementary Information:**

The online version contains supplementary material available at 10.1007/s00216-023-04610-0.

## Introduction

New demands for analytical chemistry are emerging today to address increasing issues such as global environmental concerns. To meet these problems, environmentally friendly and sensitive analytical methods for monitoring pollutants are needed. One of the 10 substances or groups of chemicals that the World Health Organization considers to be particularly dangerous to human health is mercury. Serious health effects can result from mercury exposure, even at small doses [1]. The chemical form of mercury, the dosage, the exposed person’s age and health, the length and type of exposure (e.g., inhalation, ingestion), and other factors have a role in determining the negative consequences [2]. We may include harm to the gastrointestinal tract, neurological system, kidneys, respiratory failures, and issues with organ development in utero as some of the most important health repercussions [1, 3]. As a result of human activity and environmental processes, mercury is currently regarded as one of the most significant hazardous substances released into the atmosphere and environment. The use of mercury in pharmaceutical, paper, electrochemical, and agricultural activities is one of the primary anthropogenic sources of mercury in the environment [4]. As a result, it is currently present in both organisms and environmental matrices [5]. Mercury is typically found as a variety of compounds: (i) soluble compounds (i.e., particularly mercury ion creating aquocomplexes, organometallic, or halides); (ii) insoluble mercury (e.g., HgS and HgSe); and (iii) volatile mercury species (e.g., organometallic, elemental mercury and inorganic compounds) [6]. The group of chemicals known as organometallic mercury compounds, in which mercury (Hg) is bonded to one or two carbon atoms, is among the most hazardous [7]. These compounds are exceedingly dangerous to a variety of living organisms because of their propensity to accumulate in the environment and their attraction to the sulfur thiol groups (-SH) of proteins and lipid tissues [8]. Similar to its organometallic compounds, mercury ion has an affinity for the thiol groups in proteins and may bioaccumulate in living organisms [9]. Due to their high toxicity and growing environmental impact over the past few decades, the classification of these substances is crucial [10]. Gas chromatography (GC) or liquid chromatography (LC) are often used to separate mercury species prior to their detection. Organomercury molecules, both volatile and non-volatile, may be separated using LC in a variety of applications. Spectrophotometry, fluorescence, and plasma-based spectrometry are the three major categories of detection technologies utilized in conjunction with LC [6, 11]. The extensive studies performed by researchers involving the speciation of mercury using LC are reviewed by Harrington [12].

Because of the trace levels of mercury species in environmental samples, preconcentration procedures are typically required to their determination. Dispersive liquid–liquid microextraction (DLLME) was introduced in 2006 by Assadi et al. [13], and it is an effective extraction method that involves distributing the immiscible extractant phase within the sample with the help of a dispersant agent (e.g., organic solvent) in order to increase the contact area between the two phases, thereby favoring analyte extraction [14]. After centrifugation, the enriched extractant phase is separated and collected for further analysis [15]. DLLME has several advantages, including short extraction times, high enrichment factors, simplicity of operation, low cost, and high extraction efficiencies [16, 17]. However, two main drawbacks appear when an organic solvent is employed as dispersant agent. Firstly, the partition coefficient of analytes in the extractant phase decreases, and, secondly the dispersant agent is usually a non-environmentally friendly organic solvent. As a result, there are currently non-organic solvent-based dispersant modalities, such as vortex or ultrasound-assisted DLLME [15].

Among the experimental conditions influencing DLLME, the solvents employed as extractant phase are crucial. To avoid having a harmful impact on the environment, they should adhere to the green analytical chemistry principles [18, 19]. Deep eutectic solvents (DES) have recently emerged as one of the most promising alternatives to the employment of hazardous organic solvents [20, 21]. DES are eutectic mixtures of two or more compounds that, because of interactions (i.e., hydrogen bonds and van der Walls forces) [22, 23] between their components, create a liquid eutectic mixture at temperatures lower than the melting points of the constituent compounds [24], being most of them non-toxic and eco-friendly extraction solvents [25, 26]. DES are typically composed of a hydrogen bond donor (HBD) and a hydrogen bond acceptor (HBA) with typical molar ratios of 1:1 and 1:2 [20, 27]. Recently, an increased interest in the use of DES in microextraction processes has been experienced, in part because of its structural adaptability and broad applicability [28]. The hydrophilic/hydrophobic nature of the solvent can be customized depending on the applications [20]. Natural deep eutectic solvents, often known as NADES, are another sub-class of DES whose components are originated from nature [27, 29]. NADES possess the same physical and chemical properties than DES and all their boundaries, as low or non-toxicity, low vapor pressure, high thermal stability, simplicity of synthesis at room temperature, high purity, and low cost. On the other hand, DES are also known as cheap analogues of ionic liquids [30, 31]. This family of emerging solvents is increasingly being used for novel analytical approaches more environmentally friendly than traditional methods, which are based on harmful organic solvents [25].

Assuming all these arguments, the aim of this study was the development of a simple, inexpensive, fast, sensitive, and environmentally friendly sample preparation method based on DLLME using a NADES (DL-menthol and decanoic acid, 2:1 molar ratio) for the separation and preconcentration of mercury species at trace levels from water samples prior to speciation by LC-UV–Vis.

## Experimental

### Reagents and water samples

Methanol (MeOH), ethanol (EtOH), acetonitrile (ACN), and acetic acid (HAc) were all obtained from Scharlau Chemie, LC grade (Barcelona, Spain). Tetrahydrofuran (THF) was purchased from Sigma-Aldrich, LC grade (Steinheim, Germany). The ultrapure water used to prepare the mobile phase in the LC system (resistivity ≥ 18 MΩ cm) was obtained by a PURELAB flex 3 purification system (Elga LabWater, High Wycombe, UK). The sodium acetate (NaAc) and sodium ethylenediaminetetraacetic acid (EDTA) were purchased from Sigma-Aldrich.

Analytical standard solution of mercury (II) of 1000 mg L^−1^ in 1% HNO_3_ was purchased from High-Purity Standards (Charleston, SC, USA). CH_3_HgCl and C_2_H_5_HgCl, both from Dr Ehrenstorfer GmgH (Augsburg, Germany), were dissolved in EtOH to provide stock standard solutions of methylmercury and ethylmercury (1000 mg L^−1^). By dissolving C_6_H_5_HgCl (Dr Ehrenstorfer GmgH) in EtOH, a stock standard solution of 1000 mg L^−1^ phenylmercury was prepared. Daily, mixed standard solutions were made using proper dilutions of the stock solutions in EtOH. All solutions were kept at 4 °C in the dark. The NADES was prepared with DL-menthol (hydrogen bond acceptor) (purity 98%) from Alfa-AesarTM (Tewksbury, MA, USA) and decanoic acid (hydrogen bond donor) (purity 98%) from Sigma-Aldrich. Dithizone was obtained from Merck (Darmstadt, Germany), and 5 mg of dithizone was dissolved in 25 mL of ACN to create the working solution. To optimize the sample pH, several buffer solutions were studied. For buffering the sample at pH 4.96, a solution of HAc and NaAc was employed. For pH between 6.0 and 8.5, phosphate buffer salts (NaH_2_PO_4_/Na_2_HPO_4_, supplied by PanReac Química S.L.U (Castellar de Vallés, Spain)) were used. Finally, phosphate buffer salts (Na_2_HPO_4_/Na_3_PO_4_) also supplied by PanReac Química S.L.U were employed to adjust the pH at 11 and 12.04. Reagents were utilized without any additional purification.

Different water samples from Spain were analyzed: (i) Serpis river in Cocentaina, (ii) Albufera lake in Valencia, (iii) Cinca river in Barbastro, (iv) wastewater from Aguas Municipalizadas de Alicante, and (v) tap water from the water supply network of San Vicente del Raspeig.

### Materials and instrumentation

Twelve mL conical-bottomed glass centrifuge tubes from Análisis Vínicos S.L. (Tomelloso, Spain) and two LC Hamilton syringes of 100 and 1000 µL (100 µL, Model 1700 Hamilton and 1000 µL, Model 1001LTN Hamilton Bonaduz AG, Bonaduz, Switzerland) were used for the microextraction procedure. For pH measurements, a Crison micropH 2000 pH meter (Alella, Spain) was employed. After the microextraction method, the microdrop was deposited in 150 µL inserts from Análisis Vínicos S.L., which were contained into 2-mL chromatographic glass vials from Agilent Technologies (Santa Clara, CA, USA).

An Agilent liquid chromatograph 1260 Infinity II model from Agilent Technologies was used for the chromatographic analyses. This system included a degasser, a quaternary pump, a diode array detector device tuned at 475 nm, and an autosampler. For separation, a Kinetex® EVO C18 column (4.6 mm internal diameter × 150 mm length, 5 m particle diameter) from Phenomenex (Torrance, CA, USA) was used.

### Synthesis of hydrophobic NADES

Decanoic acid and DL-menthol were mixed to synthesize the hydrophobic NADES, which was created by simply combining DL-menthol (2 mol) and decanoic acid (1 mol) at 60 ºC inside of an argon environment and swirling the mixture until it formed a clear and homogenous liquid (usually 30 min).

### Procedure

**Fig. 1:**
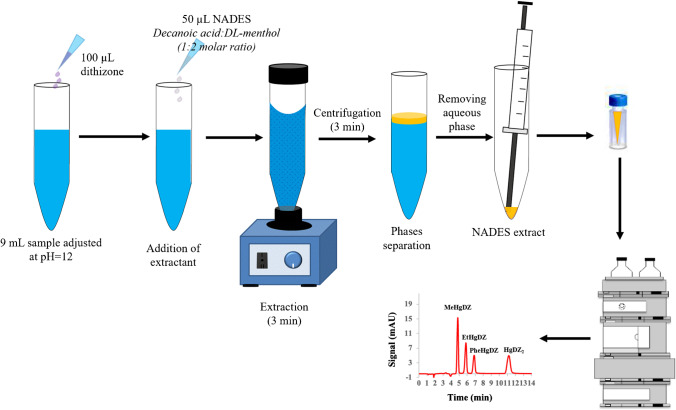
Schematic representation of the NADES-based DLLME-LC-UV–Vis procedure for preconcentration and speciation of mercury species in aqueous samples

The aqueous sample solution containing MeHg^+^, EtHg^+^, PhHg^+^, and Hg^2+^ was conditioned to pH 12 using 0.01 M phosphate buffer (Na_2_HPO_4_/Na_3_PO_4_), and 9 mL was placed in a conical-bottomed glass tube. One hundred μL of dithizone solution (200 mg L^−1^ in ACN) was added to the sample in order to complex the mercury species, and the mixture was manually shaken to ensure the correct formation of the dithizonates. Then, 50 μL of NADES (decanoic acid:DL-menthol, 1:2 molar ratio) was dropped to the sample, and the final volume was adjusted with ultrapure water to 10 mL. To guarantee NADES dispersion and analyte extraction throughout the microextraction time, the mixture was vortexed for 3 min. Subsequently, the phases were separated by centrifugation at 3000 rpm for 3 min. It is important to point out that the NADES extract formed an upper layer, being difficult to collect. Therefore, the aqueous phase was then removed using the 1000 μL Hamilton syringe. Finally, the analyte-enriched NADES (i.e., 45 µL) was then deposited into a 150-μL glass insert, contained in the 2-mL chromatographic vial, with the help of a 100 μL Hamilton syringe before being injected into the chromatographic system. Figure 1 shows a schematic representation of the general optimized NADES-based DLLME procedure. The LC injection volume was 10 µL, and the separation was carried out in isocratic mode by a mobile phase made up of THF/MeOH/(0.1 M HAc/NaAc pH 4.0 + 50 µM EDTA) (36/32/32%). In order to prevent interferences on mercury dithizonate separation from other metal ions and to avoid those ion accumulation in the chromatographic column, EDTA was added to the mobile phase [32]. The flow rate was 1.2 mL min^−1^, and the retention time values (*t*_R_) obtained for the studied compounds are provided in Table S1. Additionally, Fig. S1 displays the chromatogram registered of a real sample non-spiked and spiked at two different levels of organomercurial species (12 and 50 µg L^−1^) and mercury ion (24 and 100 µg L^−1^), after being complexed (i.e., dithizonate compounds) and DLLME extracted under the optimized conditions.

### Data processing

To determine the optimal conditions for DLLME, a two-step multivariate optimization strategy with a Plackett–Burman and a central composite design (CCD) was employed. The experimental design matrices were created, and the results were assessed using the statistical program NEMRODW® (“New Efficient Methodology for Research Using Optimal Design”) from LPRAI (Marseille, France). Each dithizonate compound peak areas recorded with LC-UV–Vis was employed as a response function for optimization.

## Results and discussion

### Characterization of hydrophobic NADES

The characterization of hydrophobic NADES and the study of their behavior were deeply carried out in a previous publication [14]. Briefly, when the synthesis of the hydrophobic NADES was done, the alcohol substituent (R1-OH) of DL-menthol (hydrogen bond acceptor (HBA)) has an interaction with the proton of decanoic acid (R_2_-CO_2_H) (hydrogen bond donor (HBD)) forming a solvent with lower melting point compared to those of the individual components. In this previous work, various ratios of DL-menthol and decanoic acid were tested by simply mixing the two components until a homogeneous mixture was obtained. These samples were analyzed showing a eutectic point for a 2:1 molar ratio of DL-menthol to decanoic acid.

### Multivariate optimization

#### Screening

Multivariate optimization approach aids in choosing the optimal value of all experimental factors involved in the extraction procedure. The Plackett–Burman design is a very useful strategy for identifying the impact on the response of multiple factors simultaneously [33]. This design, which takes into account all the factors influencing the DLLME process but does not consider the factor interaction, identifies the experimental factors with a significant effect in the extraction procedure by running few experiments and, therefore, in a more economical and environmentally friendly manner to follow the guidelines of Green Analytical Chemistry [19]. A CCD is then used to determine the optimal values for significant factors. In this work, the following independent factors were assessed: NADES volume, sample pH, chelating agent volume, extraction time, centrifugation speed, and centrifugation time. The experimental factors and levels considered in the Plackett–Burman design are shown in Table 1. Table S2 shows the experimental matrix of the six factors evaluated in twelve experiments randomly carried out using aqueous standards of 50 μg L^−1^ for organomercurial species and 100 μg L^−1^ for mercury ion and the peak areas obtained.

**Table 1:** Experimental factors and levels of the Plackett–Burman design

Factor	Level	
	Low (− 1)	High (+ 1)
NADES volume (μL)	100	200
Sample pH	7	11
Chelating agent volume (μL)	100	200
Extraction time (min)	1	3
Centrifugation speed (rpm)	2000	3000
Centrifugation time (min)	1	3

**Fig. 2:**
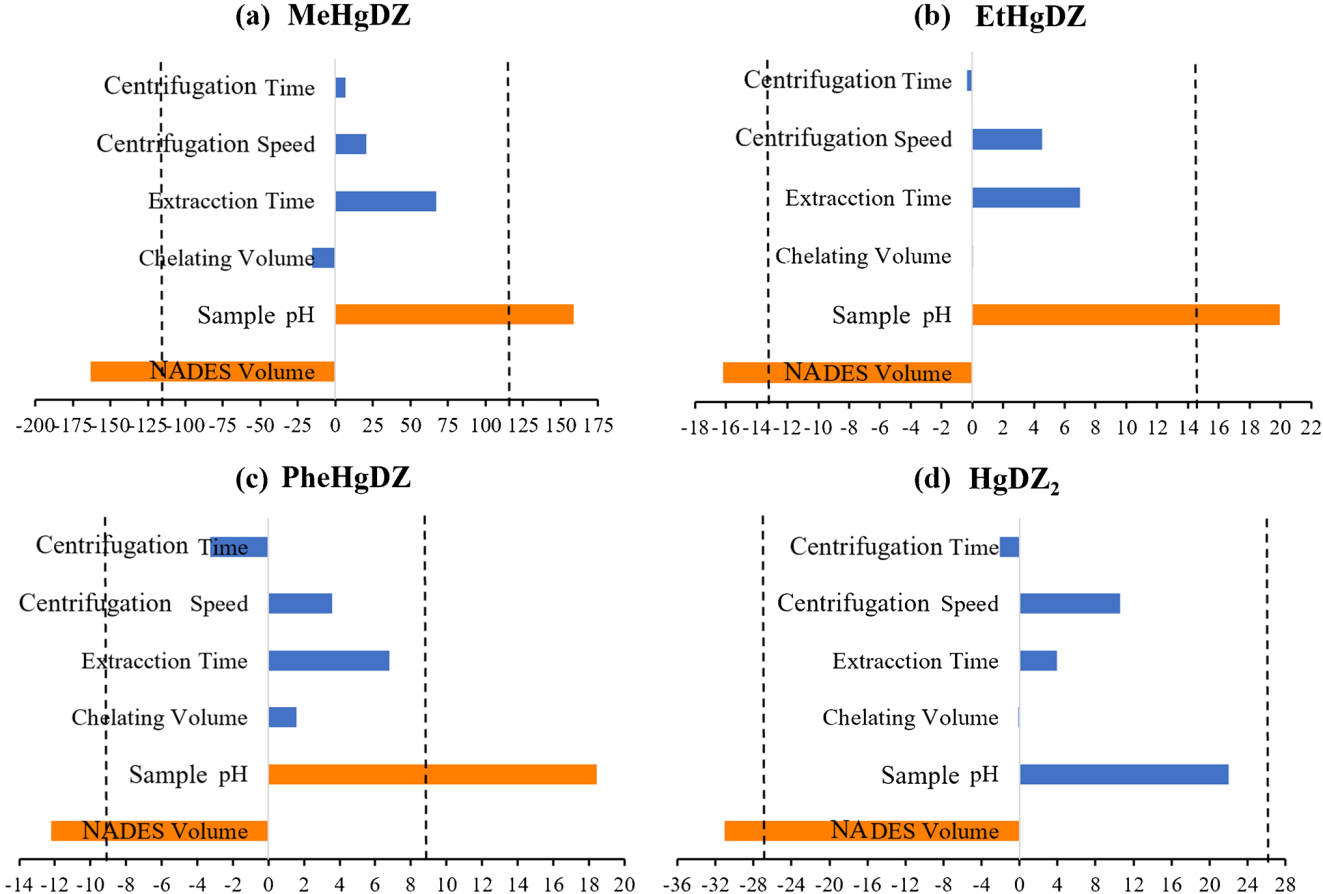
Pareto charts of the Plackett–Burman design obtained for **a** MeHgDz, methylmercury dithizonate; **b** EtHgDz, ethylmercury dithizonate; **c** PhHgDz, phenylmercury dithizonate; **d** HgDz_2_, mercury(II) dithizonate

The peak areas of the analytes of LC-UV–Vis (methylmercury, ethylmercury, phenylmercury, and mercury dithizonates) were used as response function. The data were evaluated, and the results were displayed in the Pareto chart shown in Fig. 2. The size of each bar represents the influence of the related factor and the effects that beyond the reference vertical line can be regarded as significant with a 95% probability. In Fig. 2, only the NADES volume and sample pH were reported as statistically significant factors with a 95% probability, indicating a negative influence for the NADES volume and a positive effect for the sample pH. This negative effect for the NADES volume is in line with the fact that the lower the extraction solvent volume, the higher the concentration of analytes. On the other hand, sample pH had a positive effect, and it was a significant factor for the organometallic species and non-significant on mercury ion. The formation of organomercurial complexes is promoted at higher sample pH levels, an effect that was also studied in a previous work [10]. However, the complexation of mercury ion with dithizone is insensitive to pH, as stated in other previous publications [34, 35]. Regarding the non-significant factors, the volume of complexing agent provided an excess in both values; therefore, the lower value was selected in order to reduce its use. Although the extraction time was also a non-significant factor due to the equilibrium in dispersive mode is achieved in few seconds, 3 min was selected because the cloudy solution was better formed. Centrifugation speed and time, being both non-significant factors, were selected at their higher values for a better phases separation. Therefore, the non-significant factors, namely, the volume of the complexing agent (100 µL), the extraction time (3 min), the centrifugation speed (3000 rpm), and the centrifugation time (3 min), were fixed at the most favorable level for the next optimization step.

#### Optimization

In the optimization stage, the CCD was employed. The main effects, interaction effects, and quadratic effects of the two significant factors were assessed and optimized using the surface response design (i.e., CCD). Table 2 displays the star points (± α), as well as the low, central, and high levels of the two factors optimized. Twelve experiments were randomly performed (Table S3), using aqueous standards of 50 μg L^−1^ for organomercurial species and 100 μg L^−1^ for mercury ion.

**Table 2:** Factors, star points, and low, central, and high levels studied in CCD

Factor	Level			Star points (α = 1.41)	
	Low (− 1)	Central (0)	High (+ 1)	− α	+ α
Sample pH	6.0	8.5	11	4.96	12.04
NADES volume (μL)	65	100	135	50	150

**Fig. 3:**
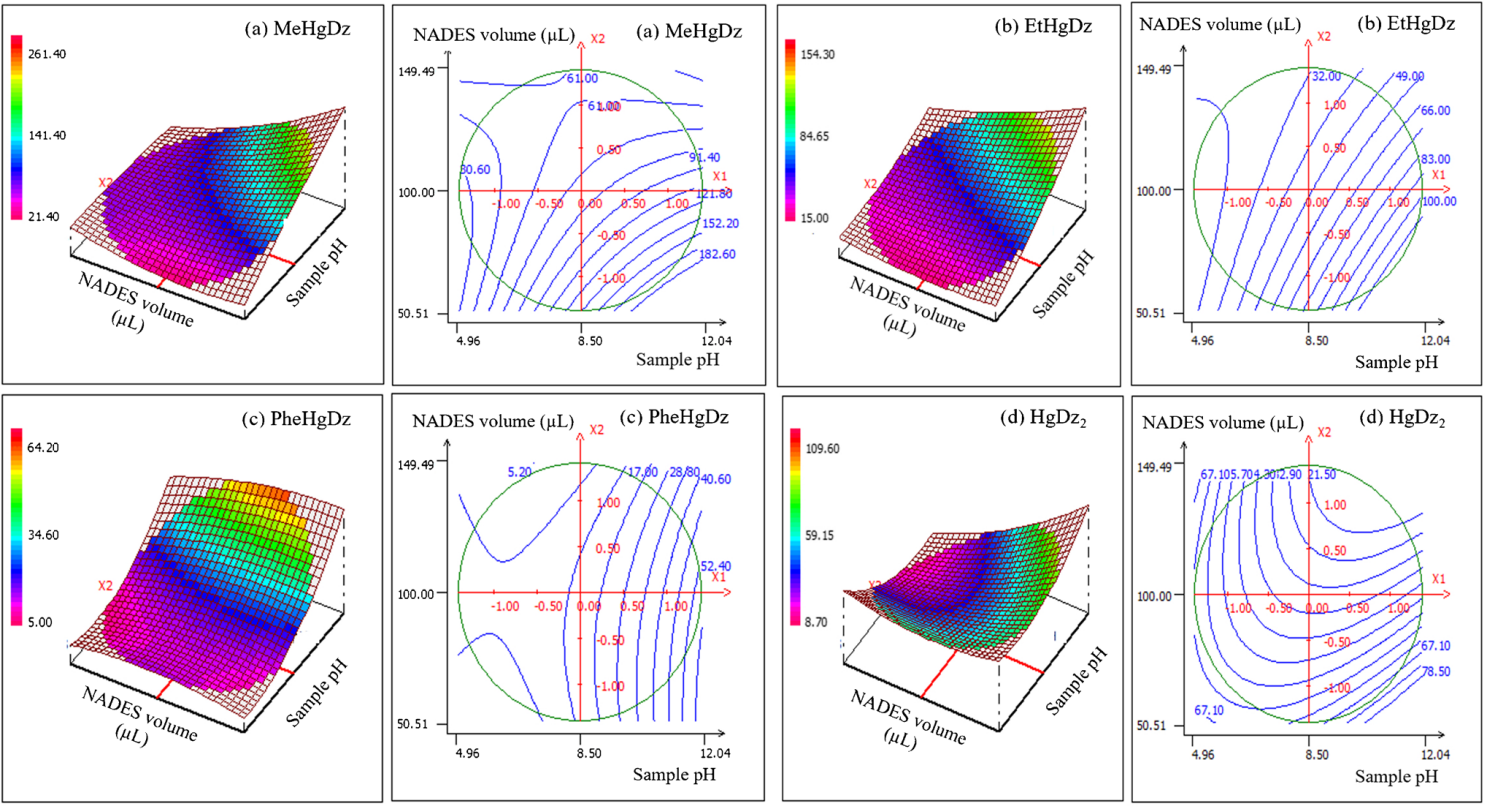
Response surfaces and contour plots of CCD obtained by plotting the NADES volume vs. pH: **a** MeHgDz; **b** EtHgDz; **c** PheHgDz; and **d** HgDz_2_

The response surfaces in Fig. 3 show that the four analytes have the same optimal values, being the lowest value of NADES volume and the highest value of sample pH. Therefore, a sample pH of 12 and a NADES volume of 50 µL were the optimal conditions for these two significant factors.

Overall, the optimal DLLME experimental conditions selected were as follows: NADES volume, 50 µL; sample pH, 12; volume of the complexing agent, 100 µL; extraction time, 3 min; centrifugation speed, 3000 rpm; and centrifugation time, 3 min.

### Analytical figures of merit

The limit of detection (LOD) and limit of quantification (LOQ), repeatability, working range, and linearity were evaluated employing aqueous standards by external calibration method to assess DLLME-LC-UV–Vis method efficiency. Table 3 shows the results. The LOD and LOQ were empirically determined by measuring progressively diluted analyte concentrations [36]. Thus, the LOD was defined as the lowest concentration at which the signal could be clearly distinguished from the blank, and the LOQ was defined as 3.3 times the LOD for each of the four analytes studied. As shown in Table 3, organomercurial compounds achieved lower limits of detection (LOD) when compared to mercury (II). The same pattern was observed in a previous study [10]. The method repeatability was investigated using six replicate experiments at two different concentration levels (25 and 50 μg L^−1^). For the four analytes, relative standard deviations (RSD) ranged from 6 to 12% at the low level and from 8 to 12% at the high level. The working range of the proposed method was established from 3 to 100 µg L^−1^ for organomercurial compounds and from 12 to 200 µg L^−1^ for mercury ion, showing good linearity with correlation coefficients ranging from 0.990 to 0.996.

**Table 3:** Main analytical figures of merit of the proposed method

Analyte	Working range (µg L^−1^)	*r*^a^	RSD^b^ (%)		LOD^c^ (µg L^−1^)	LOQ^c^ (µg L^−1^)
			25 µg L^−1^	50 µg L^−1^		
MeHg^+^	3–100	0.994 (7)	11	9	0.9	3
EtHg^+^	3–100	0.990 (7)	12	8	0.9	3
PheHg^+^	3–100	0.996 (7)	6	11	0.9	3
Hg^2+^	10–200	0.996 (7)	10	12	3	10

^a^Correlation coefficient: number of calibration points in parentheses

^b^Repeatability (*n* = 6)

^c^LOD and LOQ were empirically obtained[36]

### Comparation with other methods

The presented method employs a novel NADES derived from plant primary metabolites, which provides it renewable, sustainable, and environmentally friendly character. The use of this extractant, avoiding the use organic solvents as chlorinated compounds or ionic liquids, contributes to a greener sample preparation procedure in accordance with green analytical chemistry principles [19]. Aside from this major benefit, the proposed method has several other advantages as rapidity, the absence of dispersant agent, and the direct analysis of the enriched NADES by the chromatographic system without any additional steps. Table 4 compares this method to other mercury speciation methods that use liquid–liquid microextraction techniques for analyte preconcentration. As can be seen, the detection limits obtained in this work are similar to previous methods, with the exception of reference [37], which employs a more sensitive and expensive detector. In addition, in most of them, more hazardous extractants are used [38, 39].

**Table 4:** Comparison of different methods using liquid–liquid microextraction techniques for the speciation of mercury

Analytical method	Mercury species	Extraction time (min)	Extractant phase	LOD (μg L^−1^)
SDME-LC-UV–Vis [10]	MeHg^+^ EtHg^+^ PhHg^+^ Hg^2+^	20	Ionic liquid: [C_6_MIM][PF_6_]	1.0 1.6 7.1 22.8
DLLME-LC-ICP-MS [37]	MeHg^+^ Hg^+^	5	CCl_4_	0.0076 0.0014
DLLME-LC-UV–Vis [38]	MeHg^+^ PhHg^+^ Hg^2+^	5	Ionic liquid: [HMIM][PF_6_] Dispersant: MeOH	0.96 1.91 0.32
HF-LLLME-UV–Vis [39]	MeHg^+^ EtHg^+^ PhHg^+^	25	Na_2_S_2_O_3_	3.8 0.7 0.3
DLLME-LC-UV–Vis [This work]	MeHg^+^ EtHg^+^ PhHg^+^ Hg^2+^	3	NADES: decanoic acid:DL-Menthol (1:2)	0.9 0.9 0.9 3

*SDME-LC-UV–Vis*, single-drop microextraction combined to liquid chromatography with ultraviolet–visible detection. *HF-LLLME-UV–Vis*, hollow fiber-based liquid–liquid–liquid microextraction combined to liquid chromatography with ultraviolet–visible detection. *DLLME-LC-ICP-MS*, dispersive liquid–liquid microextraction combined with high-performance liquid chromatography-inductively coupled plasma mass spectrometry. *DLLME-LC-UV–Vis*, dispersive liquid–liquid microextraction combined to liquid chromatography with ultraviolet–visible detection

### Analysis of real water samples

The developed method was examined for the extraction and determination of organomercurials and mercury (II) in tap water, lake water, river water, and wastewater to assess its applicability. To this end, each water sample was subjected to three replicate analyses under optimal experimental conditions. The samples were filtered prior to microextraction to remove organic matter. For all water samples, the mercury species content was below the corresponding LOD values. To evaluate the matrix effects, a recovery study was carried out. Water samples were spiked before filtration at concentrations of 12 and 50 µg L^−1^ for organomercurial species and 24 and 100 µg L^−1^ for mercury ion. Table 5 shows that recovery values for methylmercury, ethylmercury, phenylmercury, and mercury ion ranged between 75 and 118% with an RSD lower than 20% with the exception of wastewater. Matrix effects were detected in this case (recoveries ranged between 45 and 110%), most likely due to the high amount of organic matter.

**Table 5:** Relative recoveries and RSD values obtained for the target analytes in the five studied real water samples

		Relative recoveries ± RSD (%)			
Sample	Spiked level^a^	MeHg^+^	EtHg^+^	PhHg^+^	Hg^2+^
Serpis river	1	98 ± 2	99 ± 15	100 ± 3	110 ± 6
	2	107 ± 13	108 ± 1	111 ± 9	105 ± 1
Tap water	1	104 ± 11	93 ± 19	90 ± 2	103 ± 1
	2	85 ± 6	103 ± 3	104 ± 4	101 ± 7
Cinca river	1	77 ± 11	76 ± 12	75 ± 7	84 ± 18
	2	106 ± 7	109 ± 9	96 ± 19	100 ± 15
Albufera	1	84 ± 6	89 ± 7	88 ± 13	81 ± 13
	2	92 ± 4	115 ± 4	109 ± 5	118 ± 5
Wastewater	1	49 ± 26	45 ± 31	54 ± 19	N.D.^b^
	2	52 ± 23	59 ± 24	77 ± 16	110 ± 22

^a^Level 1, 12 µg L^−1^ for organomercurial species and 24 µg L^−1^ for mercury ion

Level 2, 50 µg L^−1^ for organomercurial species and 100 µg L^−1^for mercury ion

^b^Not detected

## Analytical greenness metric for sample preparation

A new analytical greenness metric named AGREEprep, which is the first published metric focusing on sample preparation, has been recently suggested as a way to assess the greenness of an analytical method [40]. AGREEprep is based on ten steps of assessment that correspond to the ten principles of green sample preparation [41]. Because of prior published metrics did not pay enough attention to the sample preparation step, AGREEprep offered suitable levels of accuracy and specificity for evaluating the environmental impact of sample preparation procedures.

Scores for each of the ten individual steps in AGREEprep are ranged from 0 to 1, where the extreme values denote the poorest and best achievement, respectively. In this analytical greenness metric, each criterion has a default weight that contributes to the total score, and readers may opt to modify the default weights of each criterion and adjust them to their analytical aims, as long as they adequately justify these changes. The total score, which likewise goes from 0 to 1, where 1 denoting ideal performance, is calculated by weighting the values from each criterion. If the total score is higher than 0.5, it is considered as a green analytical method.

**Fig. 4:**
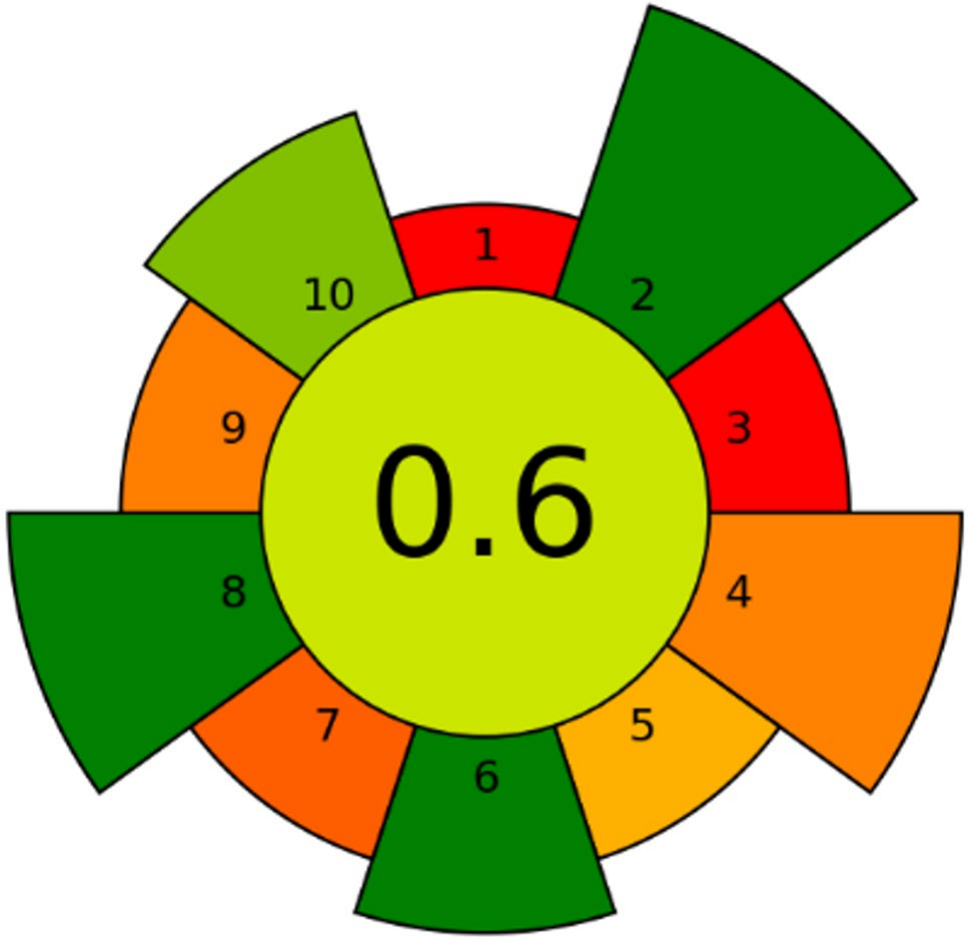
Pictogram of the analytical greenness metric for sample preparation of the proposed method

Table 6 displays the score of each criterion of the analytical greenness metric, and Fig. 4 illustrates the AGREEprep pictogram for the proposed analytical method. The visual presentation of AGREEprep enables a quick comparison of the scores of each criterion and the overall result (i.e., 0.6). The AGREEprep pictogram demonstrates a positive result that confirms that the developed method is in conformity with the current green chemistry trends by removing hazardous materials, high sample throughput, and low energy consumption and ensuring safe procedures for the operator. However, this metric indicates that there is an opportunity for improvement, particularly in the criteria of sample preparation placement, sustainability and renewability of materials, generation of waste, and the automation of the steps involved.

**Table 6:** Score of each criterion of the analytical greenness metric for sample preparation for the determination of organomercurials and mercury (II) in water by the proposed method (DLLME-LC-UV–Vis)

Criterion	Value	Score
01: sample preparation placement	Ex situ	0
02: hazardous materials	0	1
03: sustainability and renewability of materials	< 25%	0
04: waste	10 mL	0.26
05: size economy of the sample	9	0.35
06: sample throughput	100 sample h^−1^	1
07: integrate steps and automation	3 manual steps	0.19
08: energy consumption	0.63 W sample^−1^	1
09: post-sample preparation configuration for analysis	LC	0.25
10: operator’s safety	1	0.75

## Conclusions

A new, safe, inexpensive, fast, sensitive, and environmentally friendly natural deep eutectic solvent (NADES)-based analytical method has been presented for the first time for mercury speciation in water samples. A NADES (decanoic acid:DL-mentol, 1:2 molar ratio) has been used as extractant phase for mercury species separation and preconcentration prior to LC-UV–Vis separation and detection. Excellent recovery, linearity, and LOD values are obtained using affordable and commonly available instrumentation in any laboratory. Only wastewater sample shows significant matrix effects (i.e., low recovery values) most likely due to the high presence organic matter. Finally, the greenness of the method has been quantitatively evaluated using the AGREEprep metric, revealing that the combination of NADES-based DLLME with LC-UV–Vis analysis represents an acceptable green analytical method.

## Supplementary Information

Below is the link to the electronic supplementary material.

Supplementary file1 (DOCX 123 KB)
