# Supplementary material for: Natural deep eutectic solvent-based microextraction for mercury speciation in water samples
Source: Anal Bioanal Chem. 2023 Mar 6;415(18):4435–44. doi: 10.1007/s00216-023-04610-0 (PMC10328898; doi:10.1007/s00216-023-04610-0)
Supplement: Supplementary file 1 — Supplementary file1 (DOCX 123 KB) [file 216_2023_4610_MOESM1_ESM.docx]

**Electronic Supplementary Material**

**Natural Deep Eutectic Solvent-based Microextraction for Mercury Speciation in Water Samples**

Laura Ripoll, Javier Rayos, Miguel Ángel Aguirre, Lorena Vidal, Antonio Canals

*Departamento de Química Analítica, Nutrición y Bromatología e Instituto Universitario de Materiales, Universidad de Alicante, P.O. Box 99, E-03080, Alicante, Spain*

**
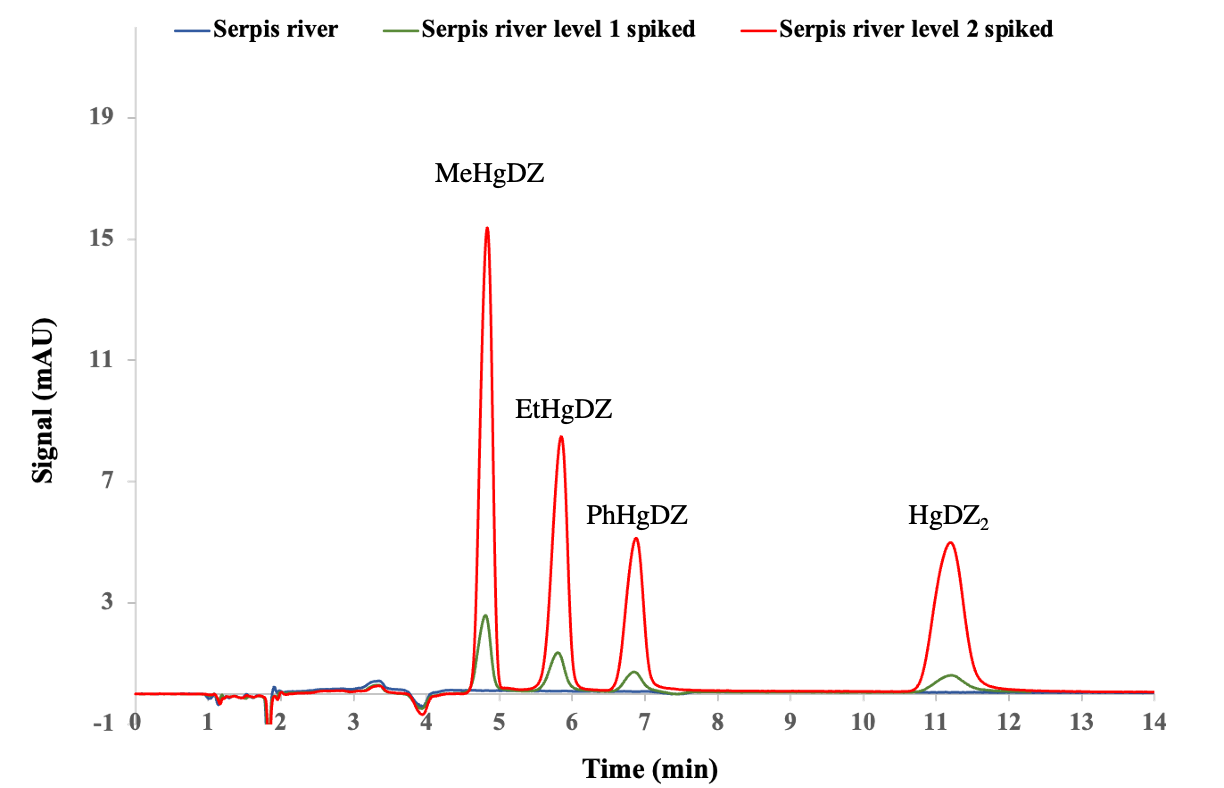
**

**Figure S1:** LC-UV-Vis chromatogram of a real sample (i.e., Serpis river) non spiked (blue line), spiked at 12 ppb for organomercurial species and 24 ppb for mercury ion (green line), and spiked at 50 ppb for organomercurial species and 100 ppb for mercury ion (red line) being DLLME under the optimized conditions. MeHgDz (Methylmercury dithizonate), EtHgDz (Ethylmercury dithizonate), PhHgDz (Phenylmercury dithizonate), and HgDz_2_ (Mercury (II) dithizonate). Wavelength = 475 nm.

**Table S1**. Retention time values of the analytes.

| Analyte | Retention time values (min) |
| --- | --- |
| MeHgDz | 4.7 ± 0.1 |
| EtHgDz | 5.8 ± 0.1 |
| PhHgDz | 6.6 ± 0.2 |
| HgDz_2_ | 11.2 ± 0.2 |

**Table S2.** Matrix of experiments of the Plackett-Burman design.

| **NADES volume**  **(μL)** | **Sample pH** | **Chelating agent**  **volume (μL)** | **Extraction time**  **(min)** | **Centrifugation speed**  **(rpm)** | **Centrifugation time**  **(min)** | **MeHgDZ**  **signal**  **(mAU)** | **EtHgDZ**  **signal**  **(mAU)** | **PheHgDZ**  **signal**  **(mAU)** | **HgDZ_2_**  **signal**  **(mAU)** |
| --- | --- | --- | --- | --- | --- | --- | --- | --- | --- |
| 200 | 11 | 100 | 3 | 3000 | 3 | 390 | 40 | 28 | 22 |
| 100 | 11 | 200 | 1 | 3000 | 3 | 813 | 92 | 62 | 142 |
| 200 | 7 | 200 | 3 | 3000 | 1 | 290 | 27 | 11 | 24 |
| 100 | 11 | 100 | 3 | 2000 | 3 | 948 | 101 | 76 | 149 |
| 100 | 7 | 200 | 1 | 3000 | 3 | 254 | 18 | 8 | 34 |
| 100 | 7 | 100 | 3 | 3000 | 1 | 535 | 41 | 16 | 53 |
| 200 | 7 | 100 | 1 | 2000 | 3 | 213 | 20 | 8 | 25 |
| 200 | 11 | 100 | 1 | 3000 | 1 | 315 | 30 | 21 | 28 |
| 200 | 11 | 200 | 1 | 2000 | 1 | 308 | 30 | 19 | 23 |
| 100 | 11 | 200 | 3 | 2000 | 1 | 736 | 77 | 70 | 84 |
| 200 | 7 | 200 | 3 | 2000 | 3 | 62 | 6 | 5 | 8 |
| 100 | 7 | 100 | 1 | 2000 | 1 | 251 | 18 | 7 | 40 |

**Table S3.** Matrix of experiments of CCD.

| **NADES volume**  **(μL)** | **Sample**  **pH** | **MeHgDZ signal**  **(mAU)** | **EtHgDZ signal**  **(mAU)** | **PheHgDZ signal**  **(mAU)** | **HgDZ_2_ signal**  **(mAU)** |
| --- | --- | --- | --- | --- | --- |
| 65 | 6.00 | 31 | 20 | 8 | 44 |
| 65 | 11.00 | 198 | 102 | 34 | 84 |
| 135 | 6.00 | 32 | 15 | 5 | 33 |
| 135 | 11.00 | 70 | 45 | 19 | 22 |
| 100 | 4.96 | 55 | 28 | 13 | 81 |
| 100 | 12.04 | 114 | 109 | 70 | 37 |
| 50 | 8.50 | 162 | 90 | 24 | 73 |
| 150 | 8.50 | 66 | 41 | 17 | 28 |
| 100 | 8.50 | 84 | 49 | 20 | 32 |
| 100 | 8.50 | 95 | 57 | 21 | 35 |
| 100 | 8.50 | 73 | 43 | 17 | 41 |
| 100 | 8.50 | 77 | 41 | 17 | 30 |
